# The Use of Digitally Assessed Stress Levels to Model Change Processes in CBT - A Feasibility Study on Seven Case Examples

**DOI:** 10.3389/fpsyt.2021.613085

**Published:** 2021-03-09

**Authors:** Miriam I. Hehlmann, Brian Schwartz, Teresa Lutz, Juan Martín Gómez Penedo, Julian A. Rubel, Wolfgang Lutz

**Affiliations:** ^1^Department of Psychology, University of Trier, Trier, Germany; ^2^Department of Psychology, University of Buenos Aires (Consejo Nacional de Investigaciones Científicas y Técnicas), Buenos Aires, Argentina; ^3^Department of Psychology, Justus-Liebig-University, Giessen, Germany

**Keywords:** ecological momentary assessments, digital phenotyping, process and outcome research, outcome monitoring, abrupt changes

## Abstract

In psychotherapy research, the measurement of treatment processes and outcome are predominantly based on self-reports. However, given new technological developments, other potential sources can be considered to improve measurements. In a feasibility study, we examined whether Ecological Momentary Assessments (EMA) using digital phenotyping (stress level) can be a valuable tool to investigate change processes during cognitive behavioral therapy (CBT). Seven outpatients undergoing psychological treatment were assessed using EMA. Continuous stress levels (heart rate variability) were assessed via fitness trackers (Garmin) every 3 min over a 2-week time period (6,720 measurements per patient). Time-varying change point autoregressive (TVCP-AR) models were employed to detect both gradual and abrupt changes in stress levels. Results for seven case examples indicate differential patterns of change processes in stress. More precisely, inertia of stress level changed gradually over time in one of the participants, whereas the other participants showed both gradual and abrupt changes. This feasibility study demonstrates that intensive longitudinal assessments enriched by digitally assessed stress levels have the potential to investigate intra- and interindividual differences in treatment change processes and their relations to treatment outcome. Further, implementation issues and implications for future research and developments using digital phenotyping are discussed.

## Introduction

The effectiveness of psychotherapy for the treatment of mental disorders has already been demonstrated in numerous meta-analyses, with outcomes comparable to and in some cases more durable than pharmacotherapy [e.g., (1–3)]. However, there is still room for improvement. Currently, about two thirds of all patients benefit from psychological treatments, yet some patients do not and 5–10% of patients even show deterioration (4). Furthermore, a significant number of patients (ranging from 18.5 to 46.5%) will experience a recurrence of their symptoms, even if they initially responded to treatment (5). These findings underline the urgency of improving psychological treatments, including cognitive behavioral therapy (CBT).

One attempt to increase the chances of treatment success for the individual patient is the call for a transdiagnostic treatment personalization [e.g., (6, 7)]. Accordingly, interventions should be personalized and adapted to each patient, consistent with patients' specific intake profiles, idiographic needs or therapists' skills [e.g., (8–12)]. This implies moving away from using treatment packages in a uniform manner and adapting CBT treatment based on patient-specific factors. Another aspect of personalization is to take a closer look at patient's' change processes^1^ over the course of treatment by repeatedly assessing outcome variables and monitoring progress [e.g., (13)]. Thereby, patients at risk of treatment failure may be identified at an early stage, which can then be reported directly back to the therapist. Monitoring therapy is particularly relevant in view of the assumption that psychotherapy progress is often non-linear and characterized by abrupt changes in symptom reduction, i.e., sudden gains (14) or sudden losses (15). Research has shown that both of these abrupt changes have a significant impact on treatment outcome. Sudden gains are associated with larger pre-post effect sizes, while sudden losses are predictive of negative outcome (16, 17). Identifying those two groups and giving feedback regarding problematic developments could help therapists adapt treatment individually (6). One example of providing personalized information to support therapists in their everyday decision-making is the Trier Treatment Navigator (TTN). Therapists are provided with personalized pre-treatment recommendations, prediction of drop-out risk, prediction of the optimal treatment strategy, a dynamic risk index to identify patients at risk for treatment failure as well as clinical problem-solving tools for personalized treatment adaptation (13, 18).

In addition to sudden symptom changes, emotional dynamics such as resistance to emotional change or inertia have been identified as potential and useful candidates to provide an early warning signal for change in depression symptoms (19). For instance, higher levels of inertia in both positive and negative affect have been found to be associated with depression and lower self-esteem (20). Furthermore, Nelson et al. (21) found higher levels of inertia in negative affect in depressed patients than in healthy controls.

One promising strategy applied to capture inertia is the use of intensive repeated measures of clinically-relevant constructs via Ecological Momentary Assessments [EMA; (22, 23)]. This method tracks participants' experiences over time in real-time and real-life situations. Self-report variables are usually collected using mobile devices several times a day and over several days. The advantages of EMA include potentially enhancing the description of within-person processes and dynamics due to overcoming retrospective biases, more frequent measurements, greater ecological validity, and increased accuracy (24). In clinical psychological research, EMA has been recently used to track a variety of patients' experiences such as perceived stress (25), symptom-related distress (26), mood and anxiety symptomatology (27), and more. Furthermore, pre-treatment fluctuations in positive and negative affect collected via EMA have been shown to predict early treatment response (28) and the prediction of patients' dropout probability has been improved using network analysis based on EMA (29). However, so far, the concept of inertia has not been extended to biological rhythms such as stress level.

To date, EMA have predominantly relied on self-report data. Recently, other sources of information have come into the picture, e.g., using passive data from personal digital devices such as smartphones to quantify moment-by-moment data. The collection of data from patients in their naturalistic settings via smartphones or other personal digital devices is defined as digital phenotyping (30). The large amount of data collected by smartphone-based digital phenotyping provides an opportunity to develop precise disease phenotypes or diagnostic markers (30) and to enhance EMA (31). Since physical activity, heart rate variability, and sleep are often associated with health outcomes, recent studies have focused on using digital phenotyping to examine their significance in psychotherapy (32, 33). For instance, Jacobson et al. (34) used actigraphy data to identify participants' diagnostic group, i.e., major depressive disorders and bipolar disorders, due to their specific and notable patterns of movement and light exposure. While depressed patients mostly showed decreased activity levels, increased levels of activity were found in patients with bipolar disorder (34). Besides identifying diagnostic groups, Jacobson and Chung (35) used passive sensor data from smartphones and wearable sensors to predict major depressive disorder severity and changes in severity across days and weeks. In view of the results and conclusions of the above-mentioned studies, it can be assumed that the integration of digital phenotyping will provide useful contributions to psychotherapy research. Nevertheless, there has been little research on how individual change in digital phenotypes (e.g., stress level) could enhance the investigation of change processes and their relation to treatment outcome.

The aim of the present feasibility study was to examine whether digitally assessed stress levels via EMA can be a valuable tool to investigate change processes during CBT. A recent model to detect both gradual and abrupt changes (36) in biological inertia is applied to passive stress data to detect individual differences. In addition, the relationship between assessed stress levels and outcome measures is being investigated to examine the predictive validity of the digital parameter.

## Methods

### Participants and Study Design

**Figure 1:**
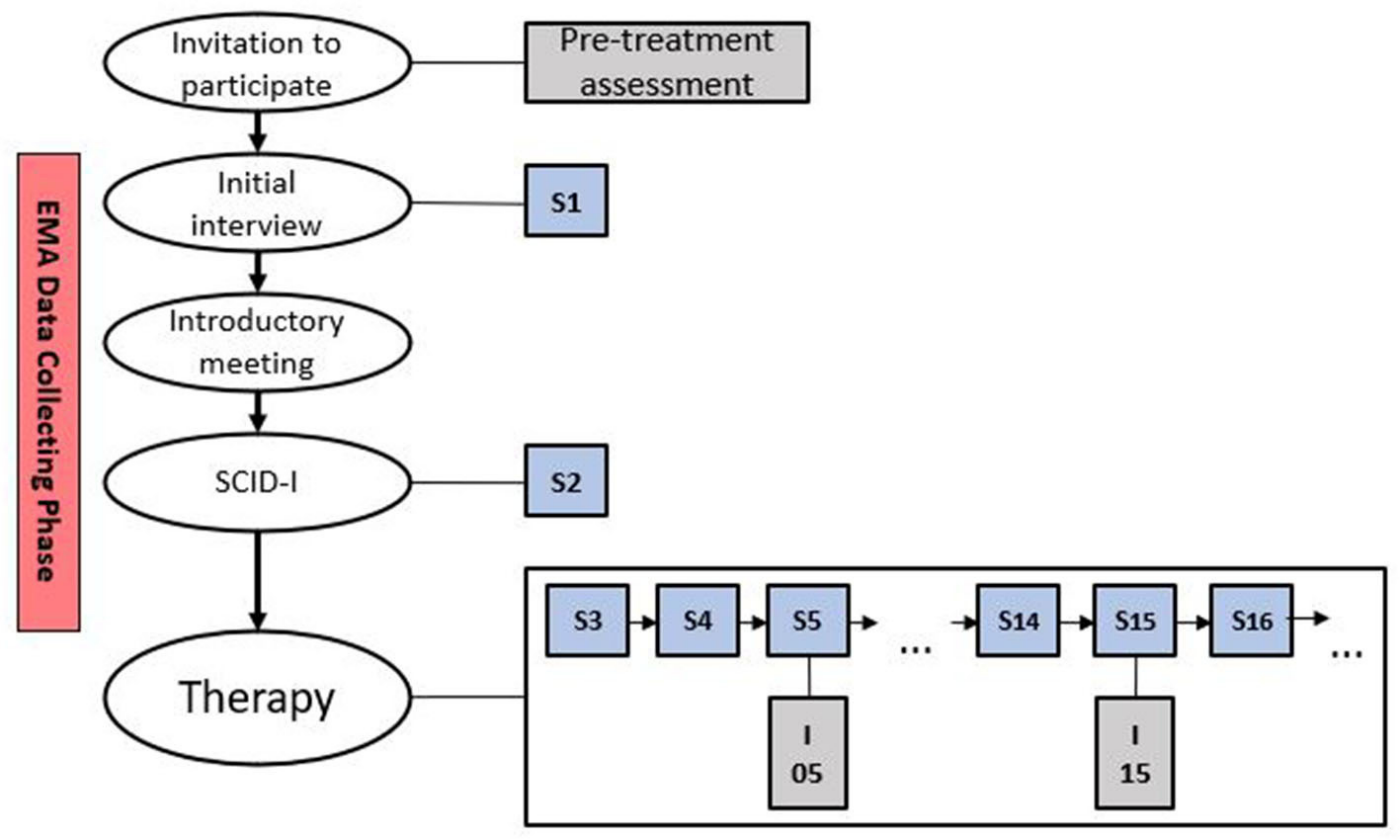
Study design. S, session; SCID-I, Structured Clinical Interview for DSM-IV-TR Axis I Disorders; I, intermediate measures.

The sample consisted of seven patients who started CBT treatment between December 2019 and March 2020 in the outpatient clinic of the University of Trier. The two-week EMA period was integrated into the clinic's regular care process and took place within the diagnostic phase. All patients filled out pre-treatment questionnaire packages, along with questionnaires every fifth session as a part of the clinic's routine assessment. A detailed description of the pre-treatment and the progress assessments can be found in the measures section, while Figure 1 is portraying the study design. In addition, the Structured Clinical Interview for DSM-IV-TR Axis I Disorders [SCID-I; (37)] was conducted by trained therapists to assess diagnoses during the diagnostic phase.

The invitation to participate in the study, detailed patient information, a declaration of consent and terms of use were sent to the patients by mail upon agreement to the regular initial interview appointment, which was conducted by experienced psychotherapists in training or licensed CBT therapists. During the initial interview, willingness to participate in the study, the exclusion criteria, and the acute need for treatment were clarified. Exclusion criteria for study admission were (a) current suicidal tendencies, (b) current mania and (c) current psychosis. All patients who did not meet the exclusion criteria were invited to participate in the study. Before the study, each patient was informed that he or she can stop the study at any time without giving reasons and without suffering any disadvantages. Following the regular initial interview, patients who agreed to participate in the study received an introductory meeting (see Figure 1). Here, the participants were handed out the fitness tracker, the app was installed and linked to the fitness tracker, furthermore the handling of the tracker and the app were explained. In addition, a hotline was made available to patients in case of open questions or technical difficulties.

### Measures

#### Pre-assessment and Progress Measurements Every Five Sessions

This section describes all relevant measures that are included in the study and are part of the clinic's routine assessment. The routine assessment includes questionnaire packages before and after treatment as well as every five sessions. The Hopkins Symptom Checklist-11 [HSCL-11, (38)], an 11-item self-report inventory for the assessment of symptomatic distress, is a brief version of the Symptom Checklist-90 [SCL-90-R; (39)] that correlates highly with the global severity index of the SCL-90-R (*r* = 0.91), and has high internal consistency [α = 0.92; (37)]. The Outcome-Questionnaire-30 [OQ-30; (40)] is a 30-item self-report measure designed to assess patient outcomes during the course of therapy, which can be aggregated to create a total score. It is a short form of the OQ-45 with which it demonstrates high levels of congruence (40). The Patient Health Questionnaire-9 [PHQ-9; (41)] is a widely used, reliable and valid assessment of depression severity. It consists of nine self-reported items and is rated from 0 to 3, resulting in an overall severity score ranging between 0 and 27. The Generalized Anxiety Disorder Scale-7 [GAD-7; (42)] is a symptom-specific instrument measuring anxiety disorder severity. It consists of seven items and is rated from 0 to 3, resulting in an overall severity score between 0 and 21 (42). Additionally, socio-economic data, such as age, gender, employment, and education status, were collected.

#### EMA Variables

EMA data was collected using a fitness tracker (Garmin *vivo* smart 4) and the corresponding app (Garmin Connect) for digital phenotyping. During the 2-week period, participants were encouraged to only take off the fitness tracker to recharge it. Heart rate, stress level, intensity minutes, movement (in steps and distance), calories, sleep duration and phases such as lighter sleep, deep sleep, being awake or REM sleep were measured. Stress level was measured every 3 min (6,720 measurements) and was based on heart rate variability. To measure stress level, Garmin is using Firstbeat Technologies Ltd., which analyzes stress from heart rate measurements. To detect heart rate, Garmin is using photophlethysmography (PPG). PPG utilizes an emitter that emits light and a detector that measures how much light is reflected, to estimate heart rate. Several factors influence the reflection of light, e.g., blood arteries absorb light better than the surrounding body tissue. The intensity of reflected light rises and falls with the contracting and swelling of the arteries as the blood pulsates. To get an insight into the performance and reliability of wearable devices, several studies have compared the results of those devices with electrocardiography (ECG) chest straps that were used at the same time (43–46). Collins et al. (44) and Bent et al. (46) found accurate results for resting heart rate when investigating several devices, among others, the Garmin *vivo* smart. However, devices differed when responding to change in activity (44, 46). Pasadyn et al. (45) investigated the response of different devices during six different treadmill speeds. The Lin's concordance correlation of the Garmin *vivo* smart and the ECG was R_c_ = 0.89 (45). The heart rate variability within each monitored period serves as indicator for the calculated stress level. To detect stress, several factors must first be excluded such as physical activity, exercise movement, recovery from exercise or changes in posture (47, 48). The obtained stress level value reports the average stress level of the monitored 3 min period. Here, values of 0–25 are considered as rest or no stress, values of 26–50 as low stress, values of 51–75 as medium stress, and values of 76–100 represent high stress. Missing values occur due to physical activity or because there is not enough data to calculate the average value. Before the analysis, the EMA data were examined for suitability. The data were suitable, when participants wore the fitness tracker more than 50% of the time. The data was downloaded as CSV. First, the Garmin UTC time stamp was converted into standard Excel date-time serial numbers. Missings were coded as –1 when there was not enough data to calculate the average stress within one monitored period, and as –2 when the participant was physically active. However, the data had to be checked for further missings in the form of entire time points missing that have not been coded accordingly.

### Statistical Analysis

The calculation of inertia trough autocorrelation or by fitting an autoregressive model [e.g., (49)] brings the drawback of assuming stationarity. However, inertia is able to change over time (50). The “critical slowing down” approach examines such changes, more specifically the increase of the autocorrelation of the symptoms, and uses this as an early warning signal [e.g., (51)]. Gradual increase in autocorrelation can also be seen as an early warning signal, but since previous methods concentrated on either modeling only abrupt or only gradual changes, a method is needed that is able to detect both changes.Time-varying change point autoregressive models (TVCP-AR, 36) were employed to detect both gradual and abrupt stress level changes for each patient. The TVCP-AR model is based on the generalized additive model framework (52), which allows both intercept and slope to change gradually over time. Further, the model is also based on the structural change point model (53, 54) in which the data are divided into regimes before and after change points (CPs). The regimes differ only in the value of the intercept, which can be extended to differences in autoregressive effects. Hamilton (53, 54) uses a transition matrix to describe the probability of moving from one regime to another for each time point. The combination of these models results in the TVCP-AR model, which allows both gradual and sudden changes in the dynamics. As the exact locations of CPs for our cases were unknown, all possible options had to be considered and an exploratory search was conducted in accordance with Albers and Bringmann (36). To find sudden changes, two models were fit to the data of each patient, one model that assumes a gradual course and one that considers a CP. After fitting the model assuming gradual change to the data and denoting the corresponding Akaike Information Criterion (AIC), the second model considering CPs was fitted and the AIC value denoted. Then, the AIC value of the gradual change model was subtracted from the AIC value of the model including a CP. If results showed no or only a small AIC improvement, there was no indication of a CP. As a threshold, we chose −15 to avoid too many false positives, which is in accordance with Albers and Bringmann (36). When two CPs are too close to each other, it implies that the number of measurements between the two CPs are too low to obtain robust estimates. It is not possible for one regime to have only one measurement. CPs that are too close to boundaries of certain intervals are difficult to detect. Furthermore, a small amount of measurements within one regime hinders the next step of the TVCP-AR model, namely modeling gradual change in the autocorrelation.

Besides presenting the case examples and their gradual and abrupt changes in stress levels, exploratory analyses concerning the associations of abrupt changes with the outcome measures at session 15 were performed. First, Pearson correlations between the number of CPs resulting from the TVCP-AR models and the outcome measures as well as between stress level and outcome measures were applied. Second, to control for initial impairment, partial correlations were computed for outcome measures at session 15 with the number of CPs as well as with stress level, adjusted for the pre-treatment assessment measured with the respective instrument. All analyses were run in *R* version 3.6.2 (55) using the package *mcgv* version 1.8-33 (56).

## Results

**Figure 2:**
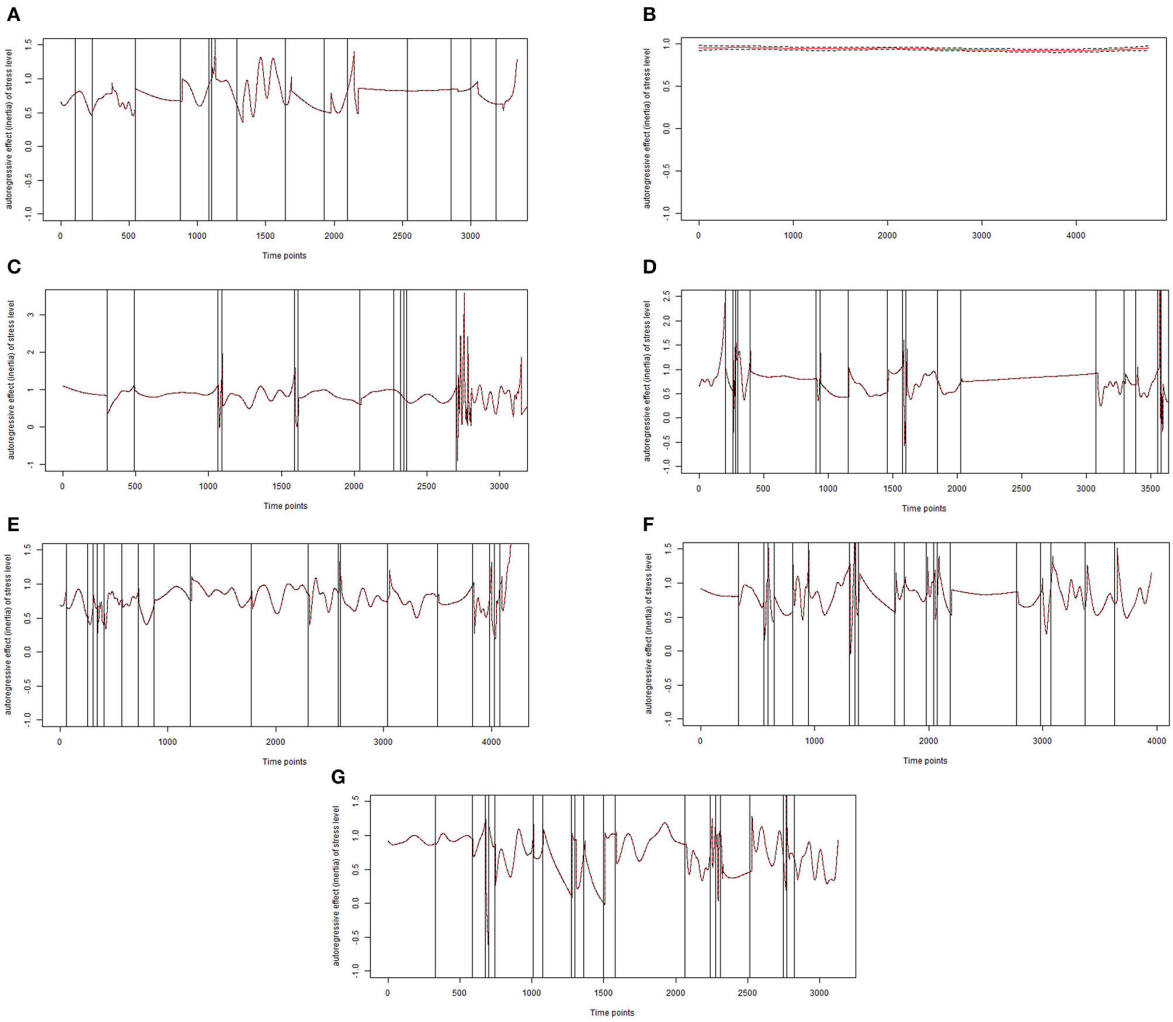
Final models of inertia for stress level for patients **(A–G)**. Each vertical line represents the exact time point of a change point.

TVCP-AR models were applied and results for the seven patients are displayed in Figure 2. Autoregressive effects of stress level are shown for each patient and CPs are marked by vertical lines. Table 1 first reports the mean values and standard deviations (SD) of the comparative sample from Lutz et al. (18) for the used outcome measures (HSCL-11; OQ-30; GAD-7; PHQ-9). Means for the pre-treatment assessment and outcome at session 15 are also portrayed for each outcome measure. In addition, product moment correlations of the number of CPs and of stress level with the outcome measures are shown. Furthermore, partial correlations controlling for initial impairment are presented. The pre-treatment assessment of every participant can be seen in Table 2, along with the session 15 assessment, which was available for six of the seven participants.

**Table 1:** Product-moment and partial correlations of number of change points, stress level and outcome measures.

		**Pre-Treatment**			**Session 15**				
			**Number of CPs**	**Stress level**		**Number of CPs**		**Stress level**	
**Outcome measure**	***M* (SD)^**a**^**	***M* (SD)^**b**^**	***r***	***r***	***M* (SD)^**b**^**	***r***	***r*_**partial**_**	***r***	***r*_**partial**_**
HSCL-11	2.20 (0.65)	2.29 (0.53)	−0.24	0.29	2.08 (0.44)	−0.18	0.08	0.13	−0.31
OQ-30	1.90 (0.56)	1.99 (0.45)	−0.48	0.52	1.87 (0.37)	−0.19	0.24	0.43	0.10
GAD-7	11.02 (5.06)	12.33 (3.27)	−0.62	0.56	12.83 (5.95)	−0.84^*^	−0.97^*^	0.68	0.55
PHQ-9	12.48 (5.73)	14.17 (4.83)	−0.58	0.66	16.00 (4.86)	−0.76	−0.64	0.57	0.01

M, Mean; SD, Standard deviation; r, Pearson product-moment correlation coefficient; r _partial_ = partial correlation; HSCL-11, Hopkins Symptom Checklist-11; OQ-30, Outcome Questionnaire-30; GAD-7, Generalized Anxiety Disorder-7; PHQ-9, Patient Health Questionnaire-9; CPs, change points;

* *p < 0.05*.

a *comparative sample from Lutz et al. (18), N = 377*.

b *N = 6, includes only patients that provided data at pre-treatment and session 15*.

**Table 2:** Outcome measures at pre-treatment and session 15.

		**Pre-treatment**				**Session 15**			
**Patient**	**Number of CPs**	**HSCL-11**	**OQ-30**	**GAD-7**	**PHQ-9**	**HSCL-11**	**OQ-30**	**GAD-7**	**PHQ-9**
A	15	2.09	2.03	9	15	1.91	2.17	8	16
B	0	2.36	2.27	16	18	2.09	1.87	22	21
C	12	2.70	2.17	14	17	2.36	1.97	16	21
D	18	2.77	2.40	15	13	2.73	1.83	15	16
E	19	2.50	1.93	12	17	1.91	2.20	10	14
F	21	3.05	2.17	14	14				
G	20	1.34	1.14	8	5	1.45	1.17	6	8

*N = 7 for pre-treatment; N = 6 for session 15; HSCL-11, Hopkins Symptom Checklist-11; OQ-30, Outcome Questionnaire-30; GAD-7, Generalized Anxiety Disorder-7; PHQ-9, Patient Health Questionnaire-9: CPs, change points*.

For the six patients, who provided data at session 15 (A, B, C, D, E, and G), results revealed higher correlations for the symptom specific instruments than for the HSCL-11 and OQ-30 (Table 1). The highest correlation of *r* = 0.84 was found between the number of CPs and GAD-7. Although only the correlation with GAD-7 was statistically significant, all outcome measures were negatively associated with the number of CPs on a descriptive level at pre-treatment assessment and at session 15. Furthermore, all outcome measures were positively correlated with stress level on a descriptive level also at both pre-treatment assessment and at session 15.

Patient A was female, 24 years old^2^, currently employed, and diagnosed with post-traumatic stress disorder (PTSD) and recurrent depressive disorder, current episode moderate. The patient reported in the initial interview a relatively high tension level, with tension quickly intensifying due to external stress factors such as conflicts at work or in social situations. With an HSCL-11 score of 2.09 and a GAD-7 score of 9, the patient was just below the average impairment of the comparison sample. However, according to the OQ-30 (2.03) and the PHQ-9 (15) she tended to score higher than the comparison sample. In the course of the first 15 sessions, the patient showed slightly reduced values in HSCL-11 and GAD-7 but also slightly higher values in OQ-30 and PHQ-9 (see Table 2). For patient A, both gradual and abrupt stress level changes were found. After excluding, the points that were too close together, a total of 15 CPs were identified during the 2-week period (Figure 2A). For example, two CPs were identified on day 4, at 17:54 (AIC difference of −18.96) and 20:21 (AIC difference of −28.83) and two CPs on day 6, at 08:39 (AIC difference of −40.97) and 08:48 (AIC difference of −40.41). The largest AIC difference found for patient A was −51.91 on day 14 at 09:21, which clearly goes beyond our threshold of −15. When looking at Figure 2, the CPs occured almost regularly except for one period of time around time point 2,200–2,800, which was during the weekend when the patient did not have to go to work.

Patient B was a currently unemployed female, 22 years old, diagnosed with recurrent depressive disorder, current episode severe without psychotic symptoms, and harmful use of alcohol. Noteworthy were the patient's compulsive behavioral tendencies to control everyday life and thus, avoid stress and the tendency to withdraw in unpleasant situations. She started the treatment with higher initial impairment scores in every outcome measure (HSCl-11, OQ-30, GAD-7, and PHQ-9) compared to the outpatient sample. In addition, the GAD-7 score at session 15 was noticeably higher (total score of 22) than at the pre-treatment assessment (total score of 16; see Table 2). The PHQ-9 also revealed higher values at session 15, however, the values for the HSCL-11 and the OQ-30 decreased. In contrast to patient A, the TVCP-AR model for patient B detected two CPs at day 1 at 14:39 (AIC difference of −18.97) and at 15:18 (AIC difference of −15.05), which are too close to each other and to the starting point, resulting in too few measurements to obtain robust results. Therefore, the final model for patient B portrays no signs of change in autocorrelation (Figure 2B), which fits the patient's tendency of avoiding any kinds of stressful situations. This example shows the most constant level of inertia compared to the other patients.

Patient C was female, 23 years old, currently employed, and was diagnosed with PTSD, an eating disorder, and recurrent depressive disorder, current episode moderate. Accordingly, the patient described handling stressful situations and tending to prevent unpleasant feelings with the help of her eating habits. She tended to be more highly impaired than the average outpatient from the comparative sample, since all of the outcome measures portrayed higher scores. Table 2 shows a slight decrease in the HSCL-11 and OQ-30 scores and a slight increase in the GAD-7 and PHQ-9 scores for patient C. Patient C showed both gradual and abrupt stress-level changes, with a total number of 12 CPs during the 2-week period (Figure 2C). For patient C, CPs also had to be excluded, as they were too close together, for example on day 5 at 15:18 (AIC difference of −17.5) and at 15:54 (AIC difference of −18.61). On day 13 at 14:48, the largest AIC difference of −33.16 was found. Notable are recurring longer phases without abrupt changes. Additionally, the level of inertia decreased over the course of the 2 weeks, toward the end more CPs were identified, and the AIC differences varied more widely.

Patient D was male, 44 years old, held a University degree and was currently employed, and diagnosed with recurrent depressive disorder, current episode moderate, and pain disorder exclusively related to psychological factors. Patient D also presented higher scores for the HSCL-11, OQ-30 and GAD-7 than the average of the outpatient sample. The PHQ-9 score was close to the average with a score of 13. He was the only patient who dropped out of treatment immediately after session 15. The outcome measures HSCL-11, GAD-7 and PHQ-9 did not reveal improvement in the course of treatment, only the score of the OQ-30 was descriptively lower at session 15. After excluding CPs that were too close together, 18 CPs were identified for patient D (Figure 2D). He displayed abrupt and gradual changes over the course of the assessment. On day 6, five jump points took place very close to each other at 18:24 (AIC difference of −28.29), at 18:39 (AIC difference of −34.63), at 18:48 (AIC difference of −53.51), at 21:15 (AIC difference of −24.76), and at 22:09 (AIC difference of −56.01), which was the largest AIC difference for this patient. The final model included CPs at 18:24 and at 22:09 on day 6. Figure 2 shows that, for example, for patients D (Figures 2C,D), besides the abrupt changes, there were also longer phases without abrupt changes compared to the other patients. Especially at the beginning of the assessment, patient D showed several CPs close to each other. However, toward the end of the assessment, a longer period of time without any CPs was observed.

Patient E was male, 25 years old, a University student, and diagnosed with recurrent depressive disorder, current episode moderate. He reported having mood swings that were associated with external stressors, e.g., work or certain social situations. The HSCL-11 score (2.50) and the PHQ-9 (17) for patient E were higher than the average of the outpatient sample. The OQ-30 and the GAD-7 scores were close to the average. The outcome measures HSCL-11, GAD-7 and PHQ-9 revealed decreased values, only the score of the OQ-30 was descriptively higher at session 15. For patient E, a gradual and abrupt pattern with 19 CPs was detected (Figure 2E). This patient displayed the largest AIC difference (−78.37) across the entire study, which was found on day 13 at 23:51. Especially at the beginning and end of the assessment period, several CPs were found quite close together. Additionally, in contrast to patients C and D, no longer periods of time without CPs were found for patient E.

Patient F was a currently employed male, 58 years old, and diagnosed with recurrent depressive disorder, current episode moderate, panic disorder without agoraphobia, and obsessive-compulsive disorder. Unfortunately, for patient F, process data assessed every fifth session were missing. Therefore, Table 2 only contains his pre-treatment assessment. He revealed the highest HSCL-11 value (3.05) of the seven patients, which was one SD higher than the average HSCL-11 outcome of the comparative sample. The remaining instruments also presented scores that were higher compared to the average of the outpatient sample. Patient F displayed the highest number of CPs (21) and showed both gradual and abrupt changes (Figure 2F). The largest AIC difference of −69.15 was located on day 12 at 07:18. Compared to patients A, B, C, and D, CPs could be found more often and quite regularly.

Patient G was female and the oldest participant (65 years old). She was diagnosed with a moderate depressive episode. Further, she portrayed the lowest scores of the seven patients at pre-treatment assessment and at session 15 on the HSCL-11, OQ-30, GAD-7, and PHQ-9 (see Table 2). All outcome measurement scores were also lower than the average of the outpatient sample, specifically one SD lower for the HSC-11, OQ-30 and PHQ-9 scores. The outcome measures HSCL-11, OQ-30 and PHQ-9 did not reveal any improvement in the course of treatment and even showed slightly higher values, only the GAD-7 score was descriptively lower at session 15. Additionally, patient G showed gradual and abrupt changes, while 20 CPs were found. The CP with the largest AIC difference (−61.85) was on day 11 at 20:45. More gradual changes were observed at the beginning, at the end, and between days 8 and 10, whereas for the rest of the assessment, many CPs were visible. It is noteworthy that patients E, F and G displayed the highest number of CPs as well as the largest AIC differences for their CPs. To summarize, stress level changed both gradually and abruptly in patients A, C, D, E, F, and G, each with varying numbers of total CPs, whereas patient B showed no signs of change. Additionally, the level of inertia varied between patients with patient B portraying the highest constant level. Finally, all patients had often or constant high levels of inertia.

## Discussion

The present feasibility study investigated whether individual differences of change patterns over time in digitally assessed stress rhythm can be detected using TVCP-AR models. The TVCP-AR model fitted two models to the data of each patient over the course of time, one model assuming a gradual course and one assuming an abrupt change point (CP). If the AIC improved when comparing the two models, this indicated the presence of a CP. When a CO was identified, the time series was split at the CP and both newly formed sections were also examined. This procedure was repeated for each new CP that was identified. Results showed abrupt changes in six of the seven participants, no change point was found in the time series of patient B. Furthermore, the number of CPs varied between the six participants. For patient A 15 CPs were identified, 12 CPs for patient C, 18 CPs for patient D, 19 CPs for patient E, 21 CPs for patient F, and finally 20 CPs for patient G. Such data collected from seven cases over a 2-week period was able to uncover individual differences in gradual and abrupt changes over time and differences in the number of CPs.

Correlations of stress level and change points with the strength and the development of patients' impairment over the course of treatment could also be shown. Although the number of patients was small, the findings suggest that the number of CPs is negatively correlated with several symptom measures, indicating that less change in physiological stress levels (i.e., inertia) tends to be associated with more self-reported symptoms. Furthermore, consistently higher stress levels correlated with higher self-reported symptoms. Specifically, the digitally assessed stress levels and the number of change points significantly correlated with the self-reported anxiety assessments via GAD-7 (at pre-treatment as well as at session 15).

These results, even so on a very limited database, are in line with previous studies that examined inertia of positive and negative affect and found higher levels of inertia to be associated with higher levels of psychological impairment, e.g., in depression and lower self-esteem as well as the onset of future symptoms (20, 21, 57). Inertia of emotional resistance has been identified as a potential candidate for an early warning signal for change in depressive symptoms (19). The results of this feasibility study suggest that physiological inertia may provide similarly useful information.

Of course, the potential of individual differences in patterns of abrupt changes in physiological and digitally assessed stress or digital phenotyping parameters should be further investigated in larger samples. Future research might benefit from taking a closer look at the patterns of individual patient differences in gradual and abrupt change over time not only related to symptoms, but also to process measures of psychotherapy. These patterns could be generated for varying parameters of change and analyzed in association with within- and between-patient change processes [e.g., (12)]. Future studies with larger samples will allow a better investigation of how those parameters can predict outcome or how they might be influenced by specific clinical techniques or strategies during treatment. Knowledge about process variables that might influence physiological inertia (or other digital parameters) could provide meaningful information on detecting mechanisms of change in psychotherapy. To increase the probability of identifying such mechanisms, change in physiological stress parameters could be investigated over a longer period of time or even for the entire duration of treatment. Finally, the quality of the physiological data collected and the psychological changes found could be further investigated by examining the relationship to psychological variables assessed simultaneously.

## Limitations and Conclusion

Digital phenotyping offers some new potential to investigate change processes in psychotherapy, however, it is at a preliminary stage and thus, several limitations have to be considered. First, as mentioned above, larger studies must be conducted to get a clearer picture of such digitally assessed parameters of inertia, their potential to investigate change processes, and their potential function as an early warning signal for negative or positive treatment outcome. One aspect that contributed to the small sample size was the first-time implementation of that particular pilot study into routine processes of the outpatient clinic. First, patients had to be made aware of the project, also there were many missings among some patients due to a lack of commitment to wear the watch more than 50% of the time. In the end, the introductory meeting was the main contributor for the small number of participants, as it took place face-to-face, which was only possible to a limited extent during the Covid-19 pandemic. Furthermore, all digital phenotyping results (e.g., stress, sleep, physical activity) depend on the accuracy of the fitness tracker used. Fitness tracker measurement errors and differences between different products need to be considered. Several studies already examined the accuracy of wearable devices measuring physiological parameters (43–46). However, more studies are required to investigate the current state of different wearables. More specifically, studies are needed that compare the performance of wearables with the performance of already validated methods, not only for heart rate or activity measured in steps, but also for sleep duration, sleep phases, and calories. Finally, one also should be aware of possible technical problems when using fitness-trackers. In order for the data from the fitness tracker to be uploaded to the server, a connection with the app must be established via Bluetooth. If participants do not establish the Bluetooth connection with the app before returning the fitness tracker, data will be lost. In addition, there are occasional missings during data transfer in the form of time points that are missing and that are not coded accordingly. This must be taken into account when cleaning the data.

One advantage of measuring digital parameters is the large amount of data that is passively measured for a longer period of time. For example, stress level was measured continuously and displayed for every 3-min section in this study resulting in a maximum of 6,720 measurements per patient. However, an issue that occurred with patients in our study related the closeness of some change points. The TVCP-AR model needs enough data to identify change points and change in small periods of time between change points that are very close to each other seem harder to identify. Therefore, the model is unable to identify the exact time point of change in autocorrelation but only gives an approximation. This might be especially a problem for parameters, which are assessed less frequently over time. Finally, several patients show autocorrelation values > 1, which could be attributed to the method since it happens mostly around change points (vertical lines, see Figure 2) and the data might still contain non-stationarity.

To conclude, this feasibility study was able to present the preliminary potential of digital phenotyping by finding individual differences in stress level inertia and connecting it with clinical as well as psychometric parameters. This is the first study to examine the inertia of digitally assessed stress levels in order to investigate fine-grained change processes in CBT. First, replication in larger samples is required. Thereafter, future research should further investigate the potential of digital phenotypes to display treatment change processes and their relation to treatment outcome. Furthermore, not only biological rhythms such as stress level should be considered as predictors or parameters of psychological change, but also other digital phenotyping candidates, e.g., activity and sleep. Additionally, the potential of digital phenotyping to predict diagnostic groups could be considered (34, 35).

An improved outcome prediction based on digitally assessed stress levels could enhance prognosis and clinical decision-making. Providing therapists with this information could support them in identifying patients at risk for poor treatment outcomes early in therapy and adapting their clinical strategies accordingly. Using this new source of information on individual change might lead to direct applications in personalized treatment and monitoring processes, e.g., by integrating it into a comprehensive feedback system and reporting this information back to the therapist (13).

## Data Availability Statement

The data analyzed in this study is subject to the following licenses/restrictions: Patients provided written, informed consent for the publication of the study. No patient was under the age of 16. Cases were modified to preserve data protection. Therefore, these cases do not represent original case vignettes. Requests to access these datasets should be directed to Miriam I. Hehlmann, hehlmann@uni-trier.de.

## Ethics Statement

The studies involving human participants were reviewed and approved by ethics commission of the University of Trier. The patients/participants provided their written informed consent to participate in this study.

## Author Contributions

MH was responsible and main contributor to the concept, writing, data management, data analyses, and data interpretation. BS contributed to writing, literature analyses, data management, preparation, and data interpretation. TL contributed to the analyses of the data and data interpretation. JG and JR consulted in the literature analyses and contributed to the writing of the manuscript. WL was contributor to several of the main concepts, writing, literature analyses, and data interpretation. All authors contributed to the article and approved the submitted version.

## Conflict of Interest

The authors declare that the research was conducted in the absence of any commercial or financial relationships that could be construed as a potential conflict of interest.

## Footnotes

^1^In the following, we will not investigate a specific psychotherapeutic process or mechanism of change. Rather, our focus is on small steps of the change process itself, measured by a psychological distress variable. In other words, we do not investigate variables, which might causally influence change, such as cognitive change or the therapeutic alliance. Instead, we investigate the within-patient change process in a fine grained way (12).

^2^In order to preserve data protection, some of the socio-demographic variables have been slightly modified.
